# Systematic development of an abbreviated protocol for screening breast magnetic resonance imaging

**DOI:** 10.1007/s10549-017-4112-0

**Published:** 2017-01-30

**Authors:** David A. Strahle, Dorothy R. Pathak, Arlene Sierra, Sukamal Saha, Catherine Strahle, Kiran Devisetty

**Affiliations:** 1 Regional Medical Imaging, PC, 2486 Nerredia Street, Flint, MI 48532 USA; 2 Department of Radiology, Michigan State University College of Human Medicine, 3346 Lennon Road, Flint, MI 48507 USA; 3 Department of Epidemiology and Biostatistics, Michigan State University College of Human Medicine, 909 Fee Road, Room B601, East Lansing, MI 48824 USA; 4 Michigan State University College of Osteopathic Medicine, 965 Fee Road, East Lansing, MI 48824 USA; 5 Department of Radiology, Michigan State University College of Human Medicine, 846 Service Road, East Lansing, MI 48824 USA; 6 Department of Surgery, Drexel University College of Medicine, 3101 Emrick Boulevard, Suite 310, Bethlehem, PA 18020 USA; 7 Department of Radiology, Michigan State University College of Human Medicine, 4100 Beecher Road, Suite A, Flint, MI 48532 USA; 8 Department of Radiation Oncology, Karmanos Cancer Institute at McLaren Flint, 4100 Beecher Road, Suite A, Flint, MI 48532 USA; 9 Department of Oncology, Wayne State University, 4100 Beecher Road, Suite A, Flint, MI 48532 USA

**Keywords:** Magnetic resonance imaging, Mammography, Breast cancer, Cancer screening, Dense breast

## Abstract

**Rationale & objectives:**

We sought to develop an abbreviated protocol (AP) for breast MRI that maximizes lesion detection by assessing each lesion not seen on mammography by each acquisition from a full diagnostic protocol (FDP).

**Materials & methods:**

671 asymptomatic women (mean 55.7 years, range 40–80) with a negative mammogram were prospectively enrolled in this IRB approved study. All lesions on MRI not visualized on mammography were analyzed, reported, and suspicious lesions biopsied. In parallel, all FDP MRI acquisitions were scored by lesion to eventually create a high-yield AP.

**Results:**

FDP breast MRI detected 452 findings not visible on mammography, including 17 suspicious lesions recommended for biopsy of which seven (PPV 41.2%) were malignant in six women. Mean size of the four invasive malignancies was 1.9 cm (range 0.7–4.1), all node negative; three lesions in two women were ductal carcinoma in situ. Nine biopsied lesions were benign, mean size 1.2 cm (range 0.6–2.0). All biopsied lesions were in women with dense breasts (heterogeneously or extremely dense on mammography, *n* = 367), for a cancer detection rate of 16.3/1000 examinations in this subpopulation. These data were used to identify four high-yield acquisitions: T2, T1-pre-contrast, T1_1.5_, and T1_6_ to create the AP with a scan time of 7.5 min compared to 24 min for the FDP.

**Conclusions:**

Our analysis of a FDP MRI in a mammographically negative group identified four high-yield acquisitions that could be used for rapid screening of women for breast cancer that retains critical information on morphology, histopathology, and kinetic activity to facilitate detection of suspicious lesions.

**Electronic supplementary material:**

The online version of this article (doi:10.1007/s10549-017-4112-0) contains supplementary material, which is available to authorized users.

## Introduction

Breast MRI has potential for use in breast cancer screening due to its increased sensitivity over mammography. Screening breast MRI has primarily been studied in women at high risk for breast cancer [1, 2] based on multiple trials [3–7] and reflected in screening guidelines from the American Cancer Society [1, 2]. Allowing for evaluation of both tumor architecture and biological activity, MRI’s application as an effective and efficient screening tool in the general population is still evolving. The application of MRI in more general screening is hindered by high cost [8], required injection of contrast, reported high rates of false positives [9], and lack of expertise in interpretation [10]. The cost for breast MRI is due, in large part, to high fixed costs for equipment acquisition, operation, maintenance, and relatively low throughput due to exam duration.

Although early efforts with the empiric selection of limited acquisitions have been proposed [11], a systematic process at defining the appropriate type and number of acquisitions is lacking. Development of such an abbreviated MRI would be both cost and time efficient without sacrificing accuracy allowing for broader utilization of this sensitive tool. This study analyzes a full diagnostic protocol (FDP) breast MRI and uses this information to identify high-yield acquisitions to develop an abbreviated protocol (AP) for general breast cancer screening.

## Materials and methods

### Subjects

From 2009 to 2011, all women who obtained their routine mammograms at a community hospital or surrounding area (film screen with computer-assisted detection) were considered for eligibility in the study. All mammogram reports contained family history of breast cancer and breast density using the breast imaging reporting data system (BI-RADS) 4th edition. Women with screening mammograms, read as BI-RADS 1, negative, or 2, benign, were considered eligible as were initially incomplete examinations (BI-RADS 0) with a final BI-RADS assessment of 1, 2, or 3 after diagnostic workup. Initial BI-RADS three assessments were also eligible who received further workup, but without recommendation for biopsy, resulting in a final BI-RADS assessment 1, 2, or 3 [12]. Women with positive mammograms (BI-RADS 4a, 4b, 4c, 5, or 6) were ineligible. Other exclusion criteria included personal history of breast cancer, prior chest radiation therapy, or any MRI contraindications. Invitations and consent forms offering a breast MRI at no charge if performed within 30 days of their mammogram were sent to 1200 women of whom 671 accepted and received FDP breast MRI exams. None of the women reported having a prior breast MRI.

### Process

MRI images for this prospective study were acquired at a community hospital and interpreted by an outside radiology institution conducting this research. Both facilities are American College of Radiology (ACR) certified centers in mammography and breast ultrasound. The reading institution is also ACR certified as a breast imaging center of excellence which includes breast MRI. Accuracy of the mammogram studies (films and reports) were verified by the MRI interpreting radiologist and available during MRI interpretation performed by one of four radiologists with breast MRI experience ranging from 6 to 12 years.

A standard FDP breast MRI was performed on a typical MRI scanner (Supplementary Table 1) using the following acquisitions: T2 (non-fat suppressed), STIR (Short-TI inversion recovery), T1-pre-contrast (T1-pre) prior to injection of contrast and T1_1_, T1_2_, T1_3_, T1_4_, T1_5_, T1_6_, and T1-high-resolution (T1_HiRes_) following contrast (subscript refers to minutes post-injection). The MRI acquisition data were post-processed on a CADstream system (Merge Healthcare, Chicago, IL) to create T1 subtraction images, maximum intensity projection images, and post-injection kinetic curves-color mapping using T1-pre and post-contrast T1_1_, T1_2_, and T1_6_ acquisitions with a threshold of 80% change in pixel intensity. All enhancing and non-enhancing lesions not detected on mammography were evaluated and a report generated by the interpreting radiologist using pre-defined MRI interpretive criteria (Table 1). In parallel, each lesion was scored by acquisition using criteria defined in Table 2 and recorded by the supervising radiologist trained technicians allowing the interpreting radiologist’s evaluation of the MRI for the dictated report to be unbiased by the applied scores. The interpreting radiologist then reviewed the applied scores and made adjustments in fewer than 2% of the cases (Tables 4, 5).

**Table 1:** Initial baseline screening breast MRI interpretive criteria

Lesion (nodule or NME)	Malignant characteristics		Benign characteristics	Action
	Plateau or washout kinetic activity	Lobulated or spiculated margins	Internal septations or normal morphologic lymph node or bright on T2	
**5 mm or less**	**Yes**	**Yes**	**NA**	**Workup**
5 mm or less	Yes	–	NA	Annual screening
5 mm or less	–	Yes	NA	Annual screening
5 mm or less	–	–	NA	Annual screening
**6 mm or larger**	**Yes**	**–**	**–**	**Workup**
**6 mm or larger**	**–**	**Yes**	**Yes or No**	**Workup**
**6 mm or larger**	**Yes**	**Yes**	**Yes or No**	**Workup**
6 mm or larger	–	–	Yes	Annual screening
6 mm or larger	Yes	–	Yes	Annual screening
6 mm or larger	–	–	–	Annual screening
**BPE - asymmetric**	**Yes or No**	**NA**	**N/A**	**Workup**
BPE - symmetric	Yes or No	NA	N/A	Annual screening

– no, *N/A* not applicable, *NME* non-mass enhancement, *BPE* background parenchymal enhancement

The bold emphasizes the requirement that futher workup is necessary

**Table 2:** Scoring criteria for the full diagnostic MRI protocol

MRI	Location	In = inside FGT Out = outside FGT None = no lesion
	Signal intensity (relative to immediate surround tissue intensity) of: T2, STIR, T1pre-contrast, T1subtraction Images*, T1high-resolution	0 = Lesion intensity equal to surrounding tissue +1 = Mild increased signal intensity +2 = Moderate +3 = Marked −1 = Mild decreased signal intensity −2 = Moderate decreased −3 = Marked decreased
	Kinetic analysis (kinetic curve)	0 = Flat or persistent curve below 80% threshold 1 = Persistent enhancement surpassing 80% 2 = Plateau enhancement surpassing 80% 3 = Enhancement with washout surpassing 80%
	Morphologic score	N = No lesion 1 = Circumscribed margins, negative other morphologic abnormalities 2 = Partial lobulated/spiculated margins or with some other morphologic abnormality 3 = Diffuse Spiculated margins or other gross morphologic abnormality
	T1_1_ vs. T1_2_ effect	Description of effect
	T1_3_/T1_4_/T1_5_ requirement	Description of requirement
MXR	FGT density-by-volume	1 = 0–24% (fatty) 2 = 25–49% (scattered) 3 = 50–74% (heterogeneously dense) 4 = 75%–100% (extremely dense)

*FGT* Fibroglandular tissue, *MXR* mammography

* Subtraction images = T1_1_ through T1_6_ minus T1pre-contrast (subscript = minutes following injection)

If a lesion did not clearly meet MRI BI-RADS 1 or 2 (there were no MRI BI-RADS three assessments), an MRI BI-RADS assessment of 4a, 4b, 4c, or 5 (recorded as a 4 or 5 in Table 4) was reported and a biopsy attempt was first made by ultrasound guidance. MRI-guided biopsy was performed only after unsuccessful ultrasound biopsy in less than 10% of the cases. The data collected for this study were evaluated for the development of the AP only after completion of the two-year data collection period.

### FDP breast MRI interpretive criteria and evaluative methods for abbreviated protocol development

The evaluative process of a lesion utilizes information from three basic aspects, i.e., morphology, signal response from each individual acquisition, and kinetic activity all based on the lesion’s conspicuity (intensity relative to surrounding tissue) as well as change in conspicuity following contrast. We hypothesized that an acquisition that provides greater visual conspicuity of lesion intensity improves characterization of morphology. We further considered histopathologic outcomes seeking individual acquisitions that best distinguish suspicious from non-suspicious lesions. As such, lesion conspicuity on a scale of −3 to +3 (Table 2) is presented in Table 4 for suspicious lesions and Table 5 as means for non-suspicious lesion types. As a general observation (Table 5), the mean intensity of each pre-contrast acquisition of suspicious lesions was less than the mean for non-suspicious lesions (substantiating that malignant lesions are often more difficult to see within non-enhanced breast tissue than benign lesions), and on post-contrast acquisitions, the reverse is observed. Thus, the ratios of relative intensities reported in Table 5, and ultimately plotted in Chart 2, were defined as the absolute value of non-suspicious lesion intensity divided by the suspicious lesion intensity for acquisitions prior to contrast and the reverse for acquisitions following contrast. This allows the majority of the ratios to be reported as values higher than 1 for visual discernment of a clinical interpretation of how these lesions present themselves. When the denominator of the ratio was 0, it was assigned a value of “25+”. Finally, we considered kinetic enhancement of each lesion and recorded kinetic information as one of four commonly used kinetic curves (Table 2).

Historically, T1_3_, T1_4_, and T1_5_ post-contrast acquisitions are used for redundancy in the event patient motion causes signal degradation of one or more of the other post-contrast T1 images. No signal degradation occurred due to motion for any of our women. Therefore, these acquisitions added no value to the development of the AP, and results from these sequences are not reported in any of the tables.

The potential for increased risk of malignancy associated with increased background parenchymal enhancement (BPE) has been recently reported [13]. The inability to evaluate each focus and lack of any standard method of recording these numerous tiny lesions prompted us to create a subjective approach to reporting BPE data. Conservatively, BPE was recorded as only one unidentified mammographic finding for each quadrant of involvement, not to exceed two lesions in each breast and, regardless of signal intensity, recorded the pattern as symmetric (not requiring biopsy) or asymmetric (requiring further workup). A lesion of concern within or adjacent to an area of BPE was evaluated separately using the interpretive criteria of Table 1.

### Statistical analyses

*T* test and its nonparametric equivalent, Wilcoxon test, were used to compare the distribution of the scores and the mean and median values for a given acquisition by comparing all suspicious lesions and all type specific non-suspicious lesions. The conclusions did not differ, and the reported *p* values are from the Wilcoxon test to account for non-normality and non-equality of variances in some comparisons [14]. All analyses were performed in SAS 9.4 (SAS Institute, Cary, NC). Two-sided *p* < 0.05 was statistically significant.

## Results

### Patient characteristics

From 2009 to 2011, 671 asymptomatic women received a FDP breast MRI (Table 3), mean age 55.7 years (range 40–80). Of these, 141 (21%) had a first-degree relative with breast cancer. No woman reported having a known *BRCA1* or −*2* pathogenic mutation.

**Table 3:** Patient characteristics

All 671 women	N (%)
Age	
40–49	194 (28.9%)
50–59	274 (40.8%)
60–69	165 (24.6%)
70–80	38 (5.7%)
Density-by-volume (*n* = 671)	
0–24% (fatty)	42 (6.3%)
25–49% (scattered)	262 (39.0%)
50–74% (heterogeneously dense)	278 (41.4%)
75–100% (extremely dense)	89 (13.3%)
Detected tumors (7)	
Clinical *T*-stage (*n* = 7)^a^	
Tis	3 (42.9%)
T1	3 (42.9%)
T2	1 (14.2%)
Clinical *N*-stage (*n* = 7)^a^	
N0	7 (100%)
Pathologic *T*-stage (*n* = 7)^a^	
TX^b^	3 (42.9%)
Tis	1 (14.2%)
T1	3 (42.9%)
Pathologic *N*-stage (*n* = 7)^a^	
NX^b,c^	5 (71.4%)
N0	2 (28.6%)

^a^Biopsy proven DCIS or carcinoma

^b^Two patients with biopsy confirmed diagnosis included the patient with 2 areas of DCIS in separate quadrants (3 lesions)

^c^Two patients with clinical N0 disease did not have axillary sampling (2 lesions)

### Lesion identification

**Fig. 1:**
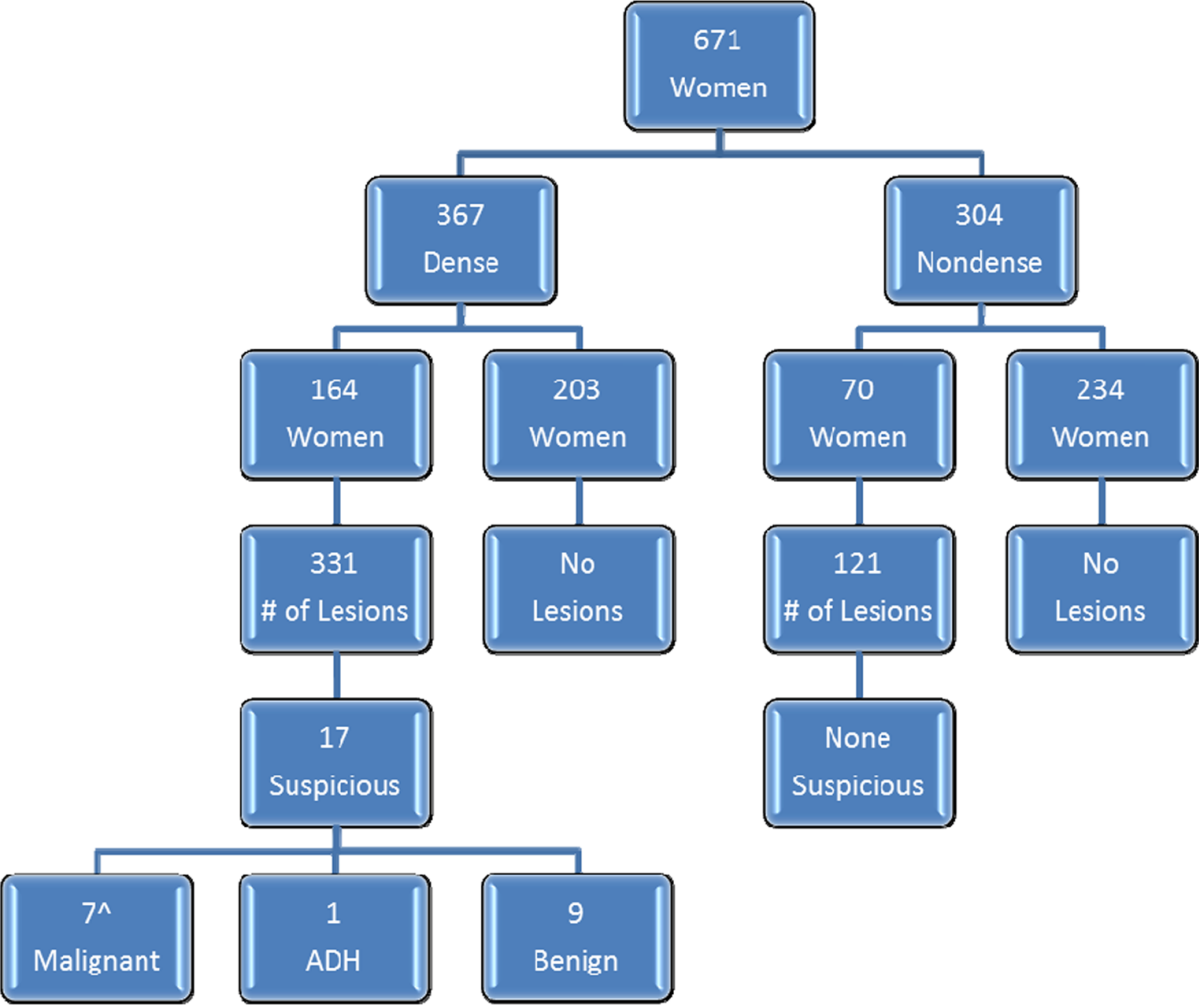
Patient and lesion distribution. * Hat* (^) one women with two positive quadrants, *ADH* Atypical ductal hyperplasia

Figure 1 provides the MRI screening outcomes. Of the 671 women, 367 (55%) had dense breasts (heterogeneously or extremely dense on mammography). Of these women, 164 (45%) had one or more lesions not detected on their mammograms, totaling 331 lesions. The remaining 304 (45%) had non-dense breasts (fatty or scattered fibroglandular tissue (FGT)) of whom 70 (23%) had one or more lesions not detected on mammography, totaling 121 lesions. Overall, as a result of obscuring FGT, 435 lesions in 218 women were not observed on mammography and assessed as MRI BI-RADS 1 or 2 (negative or benign) and 17/452 lesions (3.8%) were assessed as BI-RADS 4 or 5 (suspicious, requiring biopsy) in 16 women. None of the lesions fit the criteria for MRI BI-RADS assessment category 3.

Seventeen suspicious lesions in 16 women were biopsied (Fig. 1; Table 4) of which 7 were malignant in 6 women for a PPV3 of 41.2%. Four women were diagnosed with invasive carcinoma (mean size 1.9 cm, range 0.7–4.1), all node negative, and three lesions in two women were ductal carcinoma in situ (DCIS) (one with two areas in different quadrants) (Table 3). Although 2 of the 6 women had a final mammographic BI-RADS assessment of three prior to the MRI (one with a small nodule and the other with focal asymmetry), these mammographic findings were unrelated to the malignancies detected by MRI. All six malignancies found on MRI were among the 367 women with dense breasts for an incremental cancer detection rate of 16.3 per 1000 in this subpopulation. One additional woman, also with dense tissue, had atypical ductal hyperplasia on MRI biopsy and excision. No biopsies were prompted by MRI findings in anyone with non-dense breasts. Nine of the biopsied lesions were benign, mean size 1.2 cm, range 0.6–2.0. All pathology reports were reviewed and concordant with MRI findings.

**Table 4:** Values of suspicious (Biopsied) lesions (*n* = 17 lesions in 16 women)—scoring

	Age (years)	Location relative to FGT	T2 intensity	STIR intensity	T1-pre-contrast intensity	T1_1_ subtraction intensity	T1_2_ subtraction intensity	T1_6_ subtr action intensity	T1-high-resolution intensity	Kinetic analysis^a^ (curve)	Morphologic score	MXR density-by-volume	MXR BI-RADS	MRI BI-RADS	Size (mm)	Biopsy results
Malignant																
Lesion 1^b^	57	In	0	1	0	2	2	2	2	1	2	3	1	4	18	DCIS
Lesion 2	53	In	0	0	0	2	2	3	3	1	2	4	3	4	66	DCIS
Lesion 3	59	In	0	0	0	3	3	3	3	3	2	3	3	4	18	IDC
Lesion 4	64	In	0	2	2	3	3	1	1	2	1	3	1	4	7	IDC
Lesion 5	41	In	0	1	0	3	3	3	3	3	2	4	1	4	41	ILC
Lesion 6	53	In	0	2	0	2	2	1	1	2	2	3	2	4	8	IDC
Lesion 7^b^	57	In	0	1	0	2	2	2	2	1	2	3	1	4	20	DCIS
ADH																
Lesion 8	51	In	1	0	0	2	2	2	2	1	2	3	1	4	18	ADH
Benign																
Lesion 9	57	In	0	2	0	2	2	2	2	1	1	3	2	4	19	Fibrocystic Change
Lesion 10	40	In	−1	−1	0	2	2	1	1	1	1	4	2	4	9	Fibroadenoma
Lesion 11	54	In	0	3	0	3	3	3	3	3	1	3	2	4	7	Benign Lymph Node
Lesion 12	50	In	0	1	0	2	2	1	1	2	2	4	2	4	8	Complex cyst
Lesion 13	40	In	1	3	0	1	1	2	2	0	1	3	1	4	6	Fibroadenoma
Lesion 14	40	In	2	2	0	1	1	0	0	1	1	4	2	4	10	Sclerosing adenosis
Lesion 15	67	In	0	-2	0	3	3	1	1	3	1	3	1	4	10	Papilloma
Lesion 16	68	In	0	0	0	1	1	1	1	1	1	3	1	4	20	Papilloma
Lesion 17	52	In	0	3	0	3	3	1	1	3	2	3	1	4	20	Papilloma sclerosing adenosis fibroadenoma
Average	53	N/A	0.18	1.06	0.12	2.18	2.18	1.71	1.71	1.71	1.53	3.29	1.59	4.00	17.94	N/A
Range	40 to 68	In	−1 to 2	−2 to 2	0 to 2	1 to 3	1 to 3	0 to 3	0 to 3	0 to 3	1 to 2	3 to 4	1 to 3	4 to 4	6 to 66	N/A

Refer to Table 2 for parameter scoring definitions

*ADH* Atypical Ductal Hyperplasia, *DCIS* Ductal carcinoma in situ, *FGT* fibroglandular tissue, *IDC* Invasive ductal carcinoma, *ILC* invasive lobular carcinoma, *MXR* mammography, *N/A* not applicable

^a^Kinetic curve type was derived from T1-pre-constrast, T1_1–2_, and T1_6_

^b^Lesions 1 and 7 are from the same patient but in different quadrants

### Evaluation of lesion intensities: development of the MRI abbreviated protocol (Charts 1, 2; Table 5)

**Chart 1:**
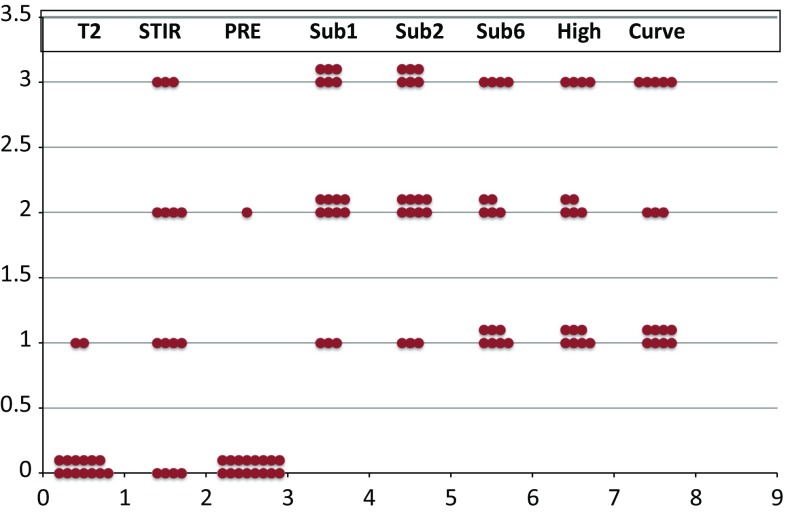
Suspicious lesions vs. intensity by acquisition

Chart 1 is a plot of intensities of every biopsied lesion demonstrating each acquisition’s ability to maximize conspicuity and thereby maximize morphologic evaluation. Based on lesion enhancement only, the subtraction images surpassed all other acquisitions in this process.

**Chart 2:**
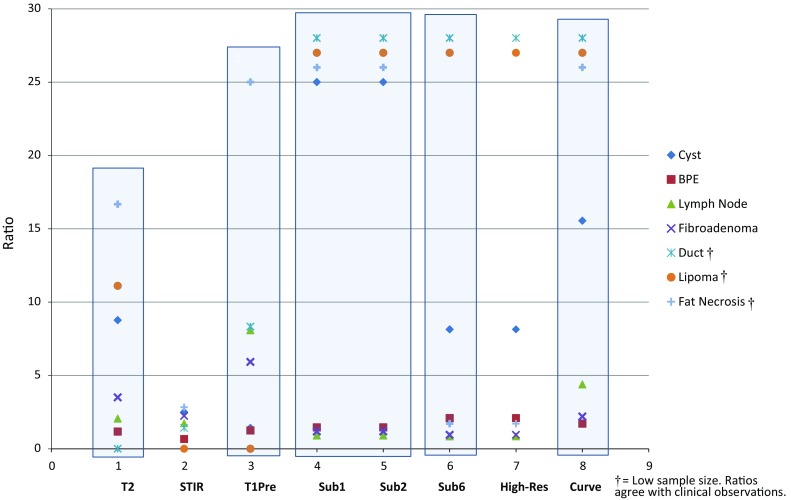
Ratios by acquisition (from Table 5)

**Table 5:** Values of non-suspicious lesions (i.e., benign, *n* = 435 lesions in 218 women)—scoring

	T2 intensity	STIR intensity	T1-pre-contrast intensity	T1_1_ subtraction intensity	T1_2_ subtraction intensity	T1_6_ subtraction intensity	T1-high-resolution intensity	Kinetic analysis^a^ (curve)	Morphologic score
Cysts (C) *n* = 186	1.58	2.66	0.17	0.05	0.05	0.21	0.21	0.11	1.06
BPE (B) *n* = 132	0.21	0.70	0.15	1.61	1.61	0.82	0.82	1.00	1.00
Lymph nodes (L) *n* = 72	−0.19	1.84	0.97	2.42	2.42	2.03	2.03	0.39	1.35
Fibroadenoma (F) *n* = 41	0.63	2.37	0.71	1.85	1.85	1.81	1.81	0.78	1.49
Dilated duct (D) *n* = 2	0.00	1.50	1.00	0.00	0.00	0.00	0.00	0.00	1.00
Lipoma (P) *n* = 1	2.00	0.00	0.00	0.00	0.00	0.00	0.00	0.00	1.00
Fat Necrosis(N) *n* = 1	3.00	3.00	3.00	0.00	0.00	1.00	1.00	0.00	1.00
Suspicious lesions (*n* = 17)^b^	0.18	1.06	0.12	2.18	2.18	1.71	1.71	1.71	1.53
RATIO between suspicious & (C)*	8.77 <0.0001	2.51 <0.0001	1.42 NS	43.60 <0.0001	43.60 <0.0001	8.14 <0.0001	8.14 <0.0001	15.55 <0.0001	1.44 <0.0001
RATIO between suspicious & (B)*	1.17 NS	0.66 NS	1.25 NS	1.35 <0.01	1.35 <0.01	2.09 <0.0005	2.09 <0.0005	1.71 <0.0001	1.53 <0.0001
RATIO between suspicious & (L)*	2.06 <0.05	1.74 <0.05	8.08 <0.001	0.90 NS	0.90 NS	0.84 NS	0.84 NS	4.39 <0.0001	1.13 NS
RATIO between suspicious & (F)*	3.50 <0.05	2.24 <0.001	5.92 <0.005	1.18 NS	1.18 NS	0.95 NS	0.95 NS	2.19 <0.0005	1.03 NS
RATIO between suspicious & (D)^c^	0.00	1.42	8.33	25+	25+	25+	25+	25+	1.53
RATIO between suspicious & (P)^c^	11.11	0.00	0.00	25+	25+	25+	25+	25+	1.53
RATIO between suspicious & (N)^c^	16.67	2.83	25.00	25+	25+	1.71	1.71	25+	1.53

Refer to Table 2 for parameter scoring definitions

*NS* statistically not significant

* p value from Wilcoxon test (<0.05 significant)

^a^Kinetic curve type was derived from T1-pre-contrast, T1_1–2_, and T1_6_

^b^From Table 4

^c^Statistical testing not applicable due to sample size; intensities and ratios agree with clinical observations

Expressed in Chart 2  (using Table 5), T2 images help differentiate cysts (8.77, *p* < 0.0001), fibroadenomas (3.5, *p* < 0.05), lipomas (11.11) and fat necrosis (16.67) from suspicious lesions, and T1-pre images help differentiate the presence of lymph nodes (8.08, *p* < 0.001), fibroadenomas (5.92, *p* < 0.005), fat necrosis (25.00) and dilated ducts (8.33). Of the three pre-contrast acquisitions, STIR images were of less utility to differentiate any non-suspicious lesion from suspicious lesions relative to either T2 or T1-pre. Clinically, these findings are supported by STIR’s inability to identify lipomas as the fat signal is suppressed. Regarding fat necrosis and cysts, STIR acquisitions added no additional information that T2 or T1-pre images did not provide. Further, the characteristic pattern of dilated ducts, observed on multiple adjacent images, is so recognizable on all other acquisitions, other than T2, that STIR acquisitions are not necessary in this regard. While visualization of lymph nodes was good on STIR images, lymph nodes were better seen on T1_1_ and T1_2_ subtraction images, and evaluation of the hila for the presence of fat was only possible on T2 images. Diagnosing fibroadenomas involves identification of internal non-enhancing septations best observed on post-contrast images, supported by the same observation on T2 images, which are not seen on STIR images. Lastly, the ability to raise suspicion for cancer by identifying a low signal surrounded by non-suppressed fat (a higher signal) on T2 images is also not possible with STIR acquisitions. Therefore, STIR acquisitions are not deemed a necessary part of the AP.

Of the post-contrast acquisitions, T1_1_ and T1_2_ subtraction images had the highest ratios in four categories—cysts (43.60, p < 0.0001), lipoma (25.00), fat necrosis (25.00), duct (25.00) (Chart 2). Further, the intensities and ratios for T1_1_ and T1_2_ subtraction images are identical to each other in all categories for both suspicious and non-suspicious lesions. Therefore, a single T1_1.5_ acquisition is sufficient to capture early lesion enhancement in place of T1_1_ and T1_2_.

T1_6_ subtraction and T1_HiRes_ were identical in their ability to differentiate the seven categories from suspicious lesions but to a lesser degree than T1_1_ and T1_2_ subtraction. Of these two acquisitions, T1_6_ is necessary for development of important kinetic curves discussed below. Further, morphologic scores (Table 5), recorded using T1_HiRes_ images, were low (range 1.00–1.49) as a result of dense tissue obscuring lesion margins and/or small lesions for which margins could not be evaluated. Also, Chart 1, T1_HiRes_ images are less intense than subtraction images for morphologic evaluation. Therefore, the T1_1.5_ subtraction image can replace the function of the T1_HiRes_ acquisition.

#### Kinetic evaluation

The importance of evaluating kinetic activity, expressed as curves and reported in Chart 2, is demonstrated by its excellent differentiating ability, associated with very low *p* values, for five of the lesion categories. The T1-pre, T1_1.5_, and T1_6_ acquisitions (used to create the curves for kinetic evaluation) were mandatory for kinetic evaluation and, consequently, proved to be important to retain in the AP.

#### BPE: a special circumstance

None of the acquisitions were of help in distinguishing BPE from a suspicious process (Chart 2). Further, kinetic evaluation is of no help as the activity of any of these tiny foci cannot be accurately determined as a result of size/volume averaging during the post-process development of kinetic curves. Therefore, the diagnosis of BPE must be on the basis of identifying the classic distribution of these tiny enhancing foci within one or both breasts and not on the basis of kinetic activity or intensity on any given acquisition.

#### The developed AP

**Fig. 2:**
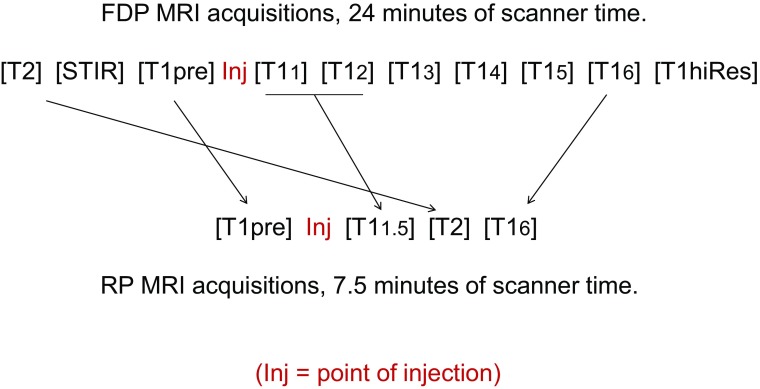
Reduction of full diagnostic protocol (FDP) to rapid protocol (RP). The subscript of the T1 acquisitions represents the time in minutes post-injection. For the FDP, the first post-injection acquisition (T1_1_) starts 35 s after initiation of the injection. For the RP, the first post-injection acquisition (T1_1.5_) starts 65 s after initiation of the injection

Our evaluative process led to the following 4 acquisitions for the AP: T2, T1-pre prior to contrast, and post-contrast T1_1.5_, and T1_6_ (necessary for kinetic curve calculation). Maintaining T2 as the first acquisition would preclude efficiency of the AP. Therefore, this acquisition, unaffected by contrast, can be placed in the time gap between T1_1.5_ and T1_6_ as the final step in the development of the AP (Fig. 2). This reduces scan time from 24 to 7.5 min. Using such an AP, all 7 malignancies and 10 suspicious benign lesions would have been identified. For institutions using T1_5_ verses T1_6_ for evaluating kinetic activity, scan time would be 6.5 min.

## Discussion

Due to overlapping tissue, lesions can be mischaracterized on mammography or missed altogether contributing to an initial PPV of recall of 4.2% (PPV1) [15]. Only after additional diagnostic imaging does PPV increase to 23.9% for biopsies recommended (PPV2) and 27.9% for biopsies actually performed (PPV3) [15]. The benefit of digital mammography over film screen is primarily limited to a subset of women of age <50 years with minimally improved PPV3 [16]. More recently, tomosynthesis has been promoted as a better screening modality. However, it only improves PPV1 to 6.4% and PPV3 to 29.2% [17] and continues use of ionizing radiation [18].

To the best of our knowledge, this is the first prospective clinical study investigating breast MRI in a general unselected female population after a negative routine mammogram that used that data to develop an AP by analyzing all lesions (suspicious and non-suspicious) missed by negative routine mammography. The findings were provocative in that all six cancers were found in the 367 women who had dense breasts at a rate of 16.3 per 1000 MRI examinations in this subset. For women with non-dense breasts, MRI did not identify any suspicious lesions. This suggests that MRI and mammography appear to serve distinct populations that could guide its future utilization.

A reported concern for screening with MRI is decreased specificity leading to increased false positives. Our standardized reading criteria (Table 1) ensured consistency and reproducibility which resulted in no repeat MRIs or supplementary imaging, other than imaging required for biopsy, for a PPV3 of 41.2%. When compared to the mammographic PPV1 of 4.2% and PPV2 of 23.9% [15], 41.2% is a large improvement for women with dense breasts and associated with a decreased rate of unnecessary biopsies. MRI improved detection of malignant lesions and better characterized a multitude of mammographically undetected benign lesions as well.

The concept of an abbreviated screening breast MRI study was recently investigated in a mild-to-moderate risk breast cancer population [11]. Even though our study was conducted at the same time as that of Kuhl et al., our approach and results differ in many respects. We evaluated all lesions (452) missed by screening mammography by each acquisition, not previously investigated in this manner, to identify the minimum required number of acquisitions for development of the AP. Kuhl et al. tested the empiric selection of only two acquisitions, one just before and one just after contrast injection without the ability to evaluate kinetic activity. Retaining kinetic/curve information is critical to accurately evaluate all lesions which also helped reduce unnecessary biopsies by nearly half. In a routine screening environment, smaller lesions would be identified in younger women, who also have more dense tissue, decreasing the ability to evaluate morphology further raising the importance of kinetic evaluation.

The standardized baseline MRI reading criteria (Table 1) allow for broader reader application, whereas Kuhl et al. stipulated their technique could only be read by breast MRI “experts.” Using kinetic data and standardized reading criteria, no woman in our study received an MRI BI-RADS assessment three (needing additional workup), whereas in Kuhl et al. 53 of 443 women, 12%, received this score. Our technique decreases uncertainty in interpretation allowing for broader application to “non-experts”. Additional facility time, scan time, cost of extra biopsies and organization resources to re-evaluate the 12% recalled in Kuhl’s et al. study, in conjunction with patient’s time and anxiety, likely outweigh the scan time advantage of 3.1 verses 7.5 min.

Multiple other centers have attempted to develop and evaluate an abbreviated MRI breast protocol; however, these were performed retrospectively, studied less generalizable populations (i.e., already had a cancer diagnosis), were not built upon a screening population in which lesions were missed on mammography, and did not analyze each independent acquisition by lesion category or evaluate the impact an abbreviated protocol might have on benign lesion identification [19–22]. Moschetta et al. studied a mixed population of patients referred for screening, problem solving, and preoperative staging, thus increasing the pre-test probability of finding cancer when imaged with MRI and obscuring the analysis [19]. Heacock et al. only utilized a population with a confirmed breast cancer diagnosis—some of which already had breast biopsy clips at the time of the MRI—thus also eliminating the ability to design an abbreviated protocol to help distinguish malignant and benign lesions if used in a screening setting [20]. Harvey et al. and Grimm et al. only studied the abbreviated MRI in a high-risk population, again limiting its applicability to a general screening environment [21, 22].

Most significantly, in these retrospective studies, there was an empiric selection of acquisitions as opposed to a more systematic approach to identify those that would have the highest yield when used on women within a screening environment. Furthermore, none of these studies identified the value and impact of breast density and its interaction with the usefulness with MRI.

Recent attention has also focused on bilateral whole breast ultrasound as a screening modality. The median ultrasound scan time in a high-risk population is reported by Berg et al. as 19 min [23], which is considerably greater than the AP MRI scan time of 7.5 min. In terms of effectiveness when studied as a screening modality in a high-risk population, Kuhl et al. reported a PPV for ultrasound of 11 versus 50% for MRI, sensitivity for ultrasound of 40 versus 91% for MRI, and specificity for ultrasound of 90.5 versus 97.2% for MRI [7]. Prospective comparisons such as this clearly demonstrate that ultrasound does not have the accuracy of MRI. This was also further reflected by Hooley et al. in which these investigators found a screening ultrasound yield in women with dense breasts of only 3.2 per 1000 women [24].

Studies suggest mammography is more sensitive for DCIS [25, 26] related to calcifications. However, another series found MRI had improved sensitivity for intermediate/high-grade DCIS over mammography [27]. DCIS not initially identified (i.e., low grade) will likely not be clinically significant or need aggressive management for which MRI screening can continue.

Even though the participants were drawn from a general population of 1200 women, only 56% accepted, which introduces the possibility of selection bias. While this rate of enrollment is similar to a prior MRI study in high-risk women [5, 28], the women willing to undergo MRI may have different baseline characteristics. Twenty-one percent had a first-degree relative with breast cancer, higher than the national average of 15% [29]. However, among the women diagnosed with breast cancer in our study, only 17% had a positive family history, similar to the national average.

At the time this study was performed, film technology was used by many facilities and considered an appropriate standard of care. Studies have since shown digital mammography can benefit certain populations [16] leading it to become the current standard. That being said, the degree of benefit from film to digital mammography is a fraction of the benefit we have demonstrated from film mammography to MRI. Compared to mammography, the benefit of MRI is most apparent in women with dense breasts, a population with well-documented challenges in all forms of mammographic imaging.

Finally, funding of the project was available for approximately 700 breast MRI examinations. Rather than apply these funds to 350 women with 350 follow-up studies of those same women, we elected to maximize the number of data points by scanning 671 women one time. Thus, a follow-up period was not part of our IRB approved study which does not allow for calculation of sensitivity, specificity, and negative predictive values.

Once our AP has been validated in an independent cohort, and used strictly in a baseline/screening environment, it will abbreviate MRI scanner time, reduce costs, and should reduce biopsies and time for reader interpretation. By retaining kinetic evaluation, our protocol allows for better lesion characterization and simplifies interpretation without detrimentally effecting overall patient/facility throughput.

Significant potential exists to improve breast cancer survival by rapid screening of women with dense breasts using this abbreviated MRI protocol and could be a supplement or even surrogate to mammographic screening of women with dense breasts, whereas mammographic technology will continue to remain the standard of care for women with fatty breasts or those that become fatty with advancing age [30].

## Electronic supplementary material

Below is the link to the electronic supplementary material.

Supplementary material 1 (DOCX 18 kb)
