# Supplementary material for: Systematic development of an abbreviated protocol for screening breast magnetic resonance imaging
Source: Breast Cancer Res Treat. 2017 Jan 30;162(2):283–95. doi: 10.1007/s10549-017-4112-0 (PMC5326631; doi:10.1007/s10549-017-4112-0)
Supplement: Supplementary file 1 — Supplementary material 1 (DOCX 18 kb) [file 10549_2017_4112_MOESM1_ESM.docx]

**SUPPORTING INFORMATION**

**SI Table 1: Full Diagnostic Protocol (FDP) Breast MRI**

| Hardware | Description |
| --- | --- |
| Type of Magnet | 1.5T VIBRANT HD (GE Medical Systems, Waukesha, WI) |
| Surface Coil | Dedicated 8 Channel HD breast array by Sentinelle, Toronto, ON |
| Breast Immobilization | Fixation plates in lateral medial direction |
| Type of Contrast Agent | Multihance (GADOBENATE DIMEGLUMINE) |
| Dose of Contrast Agent | 0.1mmol per kg body weight |
| Injection Protocol | 3 ml per second power injection, followed by 20 ml saline |

| Pulse-Sequence Parameters | T2 STIR | T2 Weighted | Dynamic Series Pre/Post-Contrast | High Resolution Post-Contrast |
| --- | --- | --- | --- | --- |
| Pulse-Sequence Type | 2D FSE-IR | 2D Frfse | 3D Vibrant Fspgr | 3D Vibrant Fspgr |
| TR/TE | 5525 ms/68 ms | 4734 ms/140 ms | Minimum | Minimum |
| Flip Angle | 180 | 90 | 10 | 10 |
| Turbo Factor | 12 | 16 | NA | NA |
| Inversion Time | 150 ms | NA | NA | NA |
| Type of fat suppression | Full | None | Special | Special |
| Orientation | Axial | Axial | Axial | Axial |
| Acquisition matrix | 384x224 | 512x384 | 448x220 | 320x256 |
| Field of view | 300-380mm | 300-380mm | 300-380mm | 300-380mm |
| No. of sections | 38-52 slices | 38-52 slices | 124-140 locs/slab | 180-250 locs/slab |
| Section thickness | 4mm | 4mm | 1.3-1.8mm | 0.8-0.9mm |
| NSA | 2 | 2 | 1 | 1 |
| Acquisition time | 3:30-5:30 min | 3:00-4:30 min | 1:00-1:05 min per dynamic series | 2:30-3:30 min |
| No. of dynamics | N/A | N/A | 1 pre-contrast; 6 post-contrast | N/A |

3D = 3 dimensional

2D = 2 dimensional

Fsgpr = Fast spoiled gradient echo

Frfse = Fast recovery fast spin echo

FSE-IR = Fast Spin Echo Inversion Recovery

N/A = Not Applicable

NSA = Number of Signal Averages

STIR = Short Tau Inversion Recovery

TR = Repetition time

TE = Echo time
